# Effect of Guizhifulingwan (Keishibukuryogan) on climacteric syndrome: study protocol for a randomized controlled pilot trial

**DOI:** 10.1186/s13063-017-1877-8

**Published:** 2017-03-21

**Authors:** Jung-Eun Kim, Junghyo Cho, Ojin Kwon, Ae-Ran Kim, Hyo-Ju Park, So-Young Jung, Joo-Hee Kim, Mikyung Kim, Hye-Yoon Lee, Jun-Hwan Lee

**Affiliations:** 1 Clinical Research Division, Korea Institute of Oriental Medicine, 1672 Yuseongdae-ro, Yuseong-gu, Daejeon 34054 Republic of Korea; 2 Department of Internal Korean Medicine, Daejeon Oriental Hospital of Daejeon University, 176-9, Daeheung-ro, Jung-gu, Daejeon 34929 Republic of Korea; 3 University of Science & Technology (UST), Korean Medicine Life Science, Daejeon, 34054 Republic of Korea

**Keywords:** Guizhifulingwan, Keishibukuryogan, Climacteric syndrome, Menopause, Randomized controlled trial

## Abstract

**Background:**

The aim of this study is to explore the efficacy of Guizhifulingwan (GFW) in the treatment of climacteric syndrome in women.

**Methods/design:**

This is a single-center, randomized, placebo-controlled, parallel-group design pilot trial. Fifty participants with climacteric syndrome will be randomly allocated to the GFW or placebo group in a 1:1 ratio. The participants will be administered GFW or placebo granules three times a day for 4 weeks and will be followed up for a further 4 weeks. The primary outcome is the mean change in menopause rating scale score at 5 weeks after randomization. Secondary outcomes include the World Health Organization quality of life-BREF scores, degrees of upward movement of qi and lower abdominal resistance and tenderness, blood stasis pattern questionnaire scores, and results of blood tests including assays for lipid profile, high sensitivity C-reactive protein, follicle-stimulating hormone, and estradiol. The feasibility outcomes include recruitment and completion rates and adherence to medication.

**Discussion:**

The results of this study will provide basic data for the design of a large-scale clinical trial for evaluating the efficacy of GFW in the treatment of climacteric syndrome in women.

**Trial registration:**

Clinical Research Information Service (CRIS), Republic of Korea, KCT0002040. Registered on 5 September 2016.

**Electronic supplementary material:**

The online version of this article (doi:10.1186/s13063-017-1877-8) contains supplementary material, which is available to authorized users.

## Background

Although menopause is a natural phenomenon in women, the accompanying pre- and post-menopausal symptoms can considerably affect their daily activities and quality of life (QOL) [1]. Although hormone replacement therapy (HRT) is known to be effective for menopausal symptoms [2], it has been reported to increase the incidence of thrombosis, stroke, and ovarian cancer [3, 4].

Because of concerns regarding the potential adverse events (AEs) associated with HRT, interest in and the use of complementary and alternative medicines related to climacteric symptoms are increasing. Although herbal medicine is among the more commonly used complementary and alternative medicines, there is little evidence supporting its efficacy [5, 6]. Guizhifulingwan (GFW; Keishibukuryogan in Japanese) has been widely used for centuries in China, Korea, and Japan for gynecological disorders caused by blood stasis [7–10]. It also has been considered effective in the management of climacteric symptoms. The symptoms for which GFW is indicated—blood stasis pattern (hot flushes with cold legs, neck ache, and stiffness), solid constitution (not in a physically weakened state), ruddy face, etc.— are not necessarily restricted to women in menopausal transition; they are generally regarded as characteristic of women suffering from vasomotor symptoms such as hot flushes and perspiration [11, 12].

Although recent clinical studies have evaluated the efficacy of GFW in the treatment of menopause-related symptoms [13–15], a randomized controlled trial (RCT) for validating the same has not yet been undertaken. Although one of the relevant studies demonstrated a positive outcome in terms of QOL in menopausal women upon treatment with GFW, the study neither included a control group nor described the criteria for blood stasis pattern during participant selection [13]. On the other hand, while a previous RCT on hot flushes in menopausal women did not yield a positive outcome in this regard, it described the importance of symptom patterns of each participant in the inclusion criteria [16]. We designed this study to better reflect the characteristics of traditional medicine by considering the traditional symptom pattern of GFW in the participant inclusion criteria and assessment methods.

## Methods/design

### Objective

The aim of this study is to explore the efficacy of GFW in the treatment of climacteric syndrome in women.

### Design

**Fig. 1:**
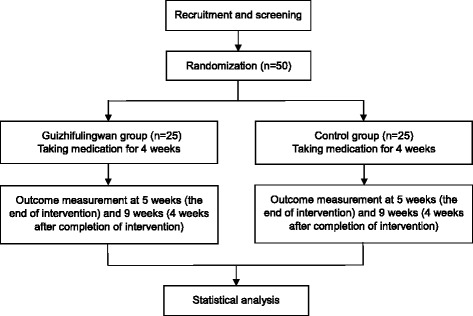
Study flow chart

**Fig. 2:**
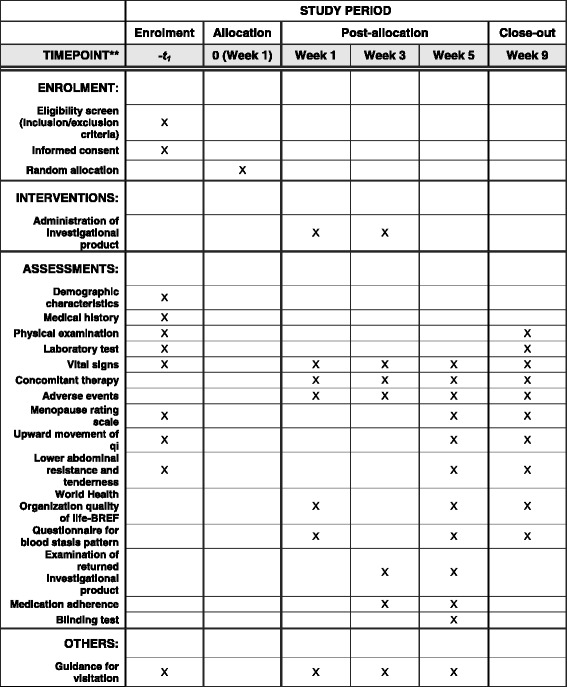
SPIRIT schedule for enrollment, interventions, and assessments

This study will be a randomized, double-blind, controlled, parallel-group pilot trial. Participants will be randomly assigned to the GFW or placebo group in a ratio of 1:1. The study period will be about 8 weeks—4 weeks each of medication and follow-up. The design is summarized in Figs. 1 and 2. The study protocol (version 1.1, 25 July 2016) follows the Standard Protocol Items: Recommendations for Interventional Trials (SPIRIT) guidelines (see Additional file 1).

### Recruitment

Participants will be recruited through advertisements in local newspapers, the subway, and our hospital.

### Participants

#### Inclusion criteria

Inclusion criteria for the study will be as follows: (1) age, 45–60 years; (2) menopausal women (including those with natural or induced menopause) with pre- and post-menopausal climacteric symptoms; (3) menopause rating scale (MRS) [17, 18] score ≥9; (4) moderate to high physical strength; (5) moderate to high degree of upward movement of qi; (6) moderate to high degree of lower abdominal resistance and tenderness; and (7) willingness to provide written informed consent.

Natural menopause is considered to have occurred after 12 consecutive months of amenorrhea with no other obvious pathological or physiological cause. The term “climacteric” encompasses perimenopause by extending the duration of symptoms for a variable period long before and after menopause. The term “perimenopause” includes the period immediately prior to menopause and the first year after menopause [19].

GFW is generally used for those who are not in a physically weakened state [10]. The degree of physical strength of inclusion criterion 4 is evaluated by referencing body physique, nutritional status, and the results of exclusion criteria 6 and 7 together, and is scored on a 3-point scale (1 = low, 2 = moderate, and 3 = high).

#### Exclusion criteria

Exclusion criteria for the study are as follows: (1) serious and unstable medical conditions; (2) severe mental disease; (3) aspartate aminotransferase (AST), alanine aminotransferase (ALT), alkaline phosphatase (ALP), and γ-glutamyl transpeptidase (γ-GTP) levels 1.5 times as high as or higher than the normal upper limit; (4) blood urea nitrogen (BUN) and creatinine (Cr) levels 1.5 times as high as or higher than the normal upper limit; (5) thyroid-stimulating hormone level 1.5 times lower than the normal lower limit; (6) body mass index <18.5; (7) hemoglobin (Hb) level <11 g/dL; (8) estrogen therapy within a year prior to the study; (9) herbal medicine therapy related to climacteric syndrome within 4 weeks prior to the study; (10) vaginal bleeding without cause after menopause; (11) lactose intolerance; (12) participation in other clinical trials; (13) residents of collective dwelling facilities such as social welfare institutions; (14) unwillingness to provide written consent; and (15) participants judged unsuitable for the clinical trial by the investigator.

### Randomization and allocation concealment

A statistician will generate random allocation numbers using a computer program (SAS [Strategic Applications Software], version 9.4; SAS Institute Inc., Cary, NC, USA). The generated numbers will be sealed in opaque envelopes and stored in double-locked cabinets. Participants who fulfill all of the inclusion criteria will be assigned to one of two groups by blocked randomization.

### Blinding

The participants, investigators, coordinators, pharmacist, monitoring agent, and statistician will be blinded to the group allocation data, which will be known only to the person in charge of random allocation. The statistician will create a random allocation table indicating assignment to group A or B. The person in charge of random allocation will deliver this allocation table and group information to the pharmaceutical company (as there is no one at the investigational products manufacturing company to create a randomization list, our team will prepare the randomization table). The pharmaceutical company will make and label investigational products based on this information. The label will include each participant’s identification number (R1001 to R1050; identical to the random allocation numbers) and visit number (V1 or V2). An opaque emergency envelope containing allocation information will be prepared and stored in a safe place in anticipation of unexpected events [20]. Violation of blinding will be considered only under circumstances where knowledge of the medication being administered to a participant is essential for treatment. The validity of blinding will be assessed according to the new blinding index [21].

### Intervention

Participants will be randomly assigned to the GFW or placebo control group in a ratio of 1:1. They will receive treatment or evaluation according to the predetermined schedule. All participants will orally ingest 3 g of granules three times a day for 4 weeks and present themselves for follow-up evaluation 4 weeks after the termination of medication. The drugs for this clinical trial will be provided to the participants twice in 2-week intervals at weeks 1 and 3. To confirm their adherence to the medication regimen, participants will be requested to return unused drugs as well as spent wrappers of used drugs.

Both GFW and the placebo are manufactured by Hanpoong Pharm. Co., Ltd. (Wanju, Republic of Korea) according to good manufacturing practice standards. Only those products that pass quality control tests will be used in this study. While GFW comprises *Ramulus cinnamomi*, *Poria*, *Moutan cortex*, *Persicae semen*, and *Radix paeoniae* [9] (3 g of GFW granules are prepared according to the granulation method using 1.33 g of GFW soft extract, which is prepared by extracting 1.33 g of each of the five herbs [same ratio] with water in an extractor, followed by filtration and concentration), the placebo comprises corn starch, lactose hydrate, citric acid hydrate, ginseng flavor powder, and caramel coloring. Both these drugs are similar in appearance and color (brown) and will be packaged in an identical shape.

### Concomitant treatment

Participants of both groups will be prohibited from concomitant use of menopause-related hormone therapy (estrogen, estrogen/progestogen, or tibolone), non-prescription medicines, functional health foods, or Oriental medicines that can potentially affect the outcome of this experiment until after the follow-up period of 8 weeks. Participants who have been on medication for chronic diseases prior to initiation of this study will be allowed to continue their regimen, and care will be taken not to alter the dosage or type of drug. Similarly, participants will be allowed to maintain their pre-trial exercise routines without altering the intensity or type of exercise. If the dosage or type of existing medication or intensity or type of exercise is changed, the participant will be required to report the same to the person in charge of this study.

### Outcome measurements

The time points for the outcome measurements are presented in detail in Fig. 2.

#### Primary outcome

The primary outcome is the mean change in MRS score at week 5. The MRS comprises 11 items encompassing 3 subscales: psychological, somato-vegetative, and urogenital domains. Each item is scored on a 4-point scale, with scores ranging from 0 (no symptoms) to 4 (very severe). Based on the total score, determined by the sum of the scores of the subscales, the degree of symptoms can be classified as follows: “no, little” (0–4), “mild” (5–8), “moderate” (9–16), and “severe” (>17) [17, 18]. The Korean version of the MRS will be used in the present study (http://www.menopause-rating-scale.info).

#### Secondary outcomes

##### World Health Organization QOL-BREF (WHOQOL-BREF)

The WHOQOL-BREF comprises 26 questions including 24 questions regarding 4 sub-sections—physical and psychological health, social relationship, and environment—and 2 questions regarding overall QOL. Each item is scored on a 5-point scale as follows: 1, strongly disagree; 2, disagree; 3, neutral; 4, agree; and 5, strongly agree. The Korean version of the WHOQOL-BREF will be used in the present study [22–24].

##### Degree of upward movement of qi

The degree of upward movement of qi is evaluated by scoring symptoms corresponding to each of five body parts on a 5-point scale (1, no symptoms; 2, mild; 3, moderate; 4, severe; and 5, extreme). The sum of these scores is divided by 5 to obtain the final value for analysis. If the final value is a decimal, it is rounded off to one decimal place. The five body parts and their corresponding symptoms are as follows: head (headache, dizziness, and heavy-headedness); face (ruddy face and hot flushes); shoulders (neck and shoulder stiffness); chest (flushing sensation and palpitation); and lower limbs (cold legs and feet). These symptoms have been identified based on previous reports [11, 25, 26], and the scoring method has been devised by the research team involved in this study.

##### Degree of lower abdominal resistance and tenderness

Abdominal examination will be performed according to the following protocol. The practitioner’s hands should be sufficiently warm before beginning the examination. The room itself should be warm enough that the participant does not shiver or become tense. The participant lies supine, comfortably, with both legs extended. When necessary, the exam can be conducted with both knees bent. The practitioner stands on the right (or left) side of the participant and first observes the entire abdomen and then palpates the lower abdomen while trying not to apply more pressure than necessary [25, 27]. The abdominal exam is performed from top to bottom and left to right. The abdominal symptoms of GFW include a resistance that feels like a palpable mass-like object in the lower abdomen. Tenderness is not severe [25, 26]. The degree of lower abdominal resistance and tenderness is scored on a 5-point scale (1, none; 2, mild; 3, moderate; 4, severe; and 5, extreme).

##### Questionnaire for blood stasis pattern

The questionnaire for blood stasis pattern comprises 14 items: sprain; contusion; old BiJeung (BiJeung shows symptoms similar to those of arthralgia and paresthesia syndrome [28]); stabbing pain; lower abdominal pain; hypochondriac pain; night pain; feeling of abdominal mass; easy congealing of blood; darkish complexion (discoloration of the face); purple and dull pallor of the mouth, lips, and tongue; bluish purple discoloration of the lower eyelid; darkish stools; and number of surgeries. Each item is scored on a 7-point scale [29].

##### Blood tests

Blood tests including those for internal lipid (low-density lipoprotein, high-density lipoprotein, total cholesterol, and triglycerides), high sensitivity C-reactive protein (for prediction of risk of cardiovascular diseases [30]), and hormone (follicle-stimulating hormone and estradiol, for understanding the climacteric condition) levels will be conducted.

#### Feasibility outcomes

##### Recruitment rate

The recruitment rate will be calculated as the percentage of participants selected from among those who undergo screening and the percentage of participants selected against the total number of participants.

##### Completion rate

The completion rate will be calculated as the percentage of participants who complete the study without interruption until the endpoint against the total number of participants.

##### Medication adherence

Medication adherence will be calculated as the percentage of doses ingested by the participants against the total dose.

#### Safety and adverse event outcomes

Adverse events (AEs) indicate undesirable and unintentional signs, symptoms, or diseases that develop after intervention during the period of a clinical trial. They do not necessarily have a causal relationship with the relevant intervention. Investigators will check participants’ vital signs and examine for manifestation of AEs, including digestive symptoms, skin conditions, and jaundice, at every visit. Investigators will also conduct physical examination and laboratory tests, including evaluation of AST, ALT, ALP, γ-GTP, BUN, Cr, Hb, and hematocrit levels and red and white blood cell, differential, and platelet counts, at the screening as well as at the fourth visit (week 9). In case of AEs, the correlation between AEs and intervention will be classified into one of the following six categories: (1) definitely related, (2) appears to be related, (3) possibly related, (4) appears to be unrelated, (5) definitely not related, or (6) unclear.

### Sample size

The null hypothesis (*H*_*0*_) of this study is that there will be no difference in the mean change in MRS scores between the GFW and placebo groups after 4 weeks of medication. The alternative hypothesis (*H*_*1*_) is the opposite scenario of *H*_*0*_. In the GFW group of a previous study [13], the mean change in MRS scores from baseline to day 30 was −7.27; the authors determined the mean change in MRS scores of the control group to be −1.45 under the assumption that it would correspond to approximately 20% of the value observed with the treatment group. With this previous study [13] as a reference, we assumed that the standard deviation of change in MRS scores from baseline to week 4 will be 6.73. With a 5% significance level and 80% power, the sample size was determined to be 21 participants per group:

$$ \mathrm{n}\kern0.5em =\kern0.5em \frac{2{\left({z}_{a/2}+{z}_{\beta}\right)}^2{\sigma}^2}{\delta^2}\kern0.5em =\kern0.5em \frac{2{\left(1.96\kern0.5em +\kern0.5em 0.84\right)}^2\kern0.5em {(6.73)}^2}{{\left(-5.82\right)}^2}\kern0.5em 20.97\approx 21 $$

Considering a 15% dropout rate, we determined that 25 participants will be ideally required per group.

### Statistical analysis

The analysis set will comprise a full analysis set (FAS) and a per-protocol set (PPS). The FAS will include participants who satisfy the intention-to-treat principle as completely as possible. The PPS will include participants who are more compliant with the study protocol [31]. The FAS will be used for the main analysis. Analysis of covariance will be performed to evaluate the values of outcomes at weeks 5 and 9 as dependent variables, the baseline values as covariates, and the group as a constant. Participants will be divided into pre- and post-menopausal groups, which will be evaluated by exploratory subgroup analysis according to MRS scores in order to determine whether the intervention is equally effective on both subgroups. Intra-group comparisons will be performed by the paired *t* test or Wilcoxon signed-rank test. The analyses will be performed considering an α-value of 5% and a power of 80%. Missing data will be imputed by multiple imputation. For socio-demographic characteristics, continuous variables will be presented as mean value and standard deviation, while categorical variables will be presented as frequency and percentage. Statistical analysis will be performed using the SAS program (version 9.4; SAS Institute Inc., Cary, NC, USA).

### Data and safety monitoring

The trial data will be saved in an electronic data capture system (Medidata Rave; Medidata Solutions Inc., New York, NY, USA). Data quality will be ensured by regular monitoring. The monitoring agent will confirm whether the data are consistent with the source documents and whether the trial is conducted according to the approved protocol. All AEs observed during the study period will be recorded and reported.

## Discussion

The purpose of this study is to explore the efficacy of GFW in the treatment of climacteric syndrome in women with blood stasis pattern. The study will also serve to monitor the safety of GFW.

Traditional Chinese medicine (TCM) has been adopted in a modified form in East Asian countries such as Japan and Korea [32, 33]. The system of pattern identification—the process of overall analysis of clinical data to determine the location, cause, and nature of disease in a patient and diagnosing a pattern/syndrome [34]—practiced in the three countries varies to a certain extent. In TCM, a practitioner recognizes the “Zheng” (clinical diagnosis based on analysis of medical history, symptoms, and signs) and confirms the treatment principle. In traditional Japanese medicine (Kampo), a practitioner chooses the most appropriate formula from among approximately 150 ready-to-use formulas, according to the “Sho” (pattern of symptoms) of the patient [32, 35]. Kampo can be characterized as a simplified and pragmatic version of Chinese herbal medicine [36]. In traditional Korean medicine (TKM), disease and pattern identification are made simultaneously as described in* Dongui Bogam* [37], which was compiled in the 17th century. TKM has been developed to include various modified methods involving the application of existing principles, methods, and formulas [38]. The diagnostic system of TKM includes the determination of diagnostic criteria based on indications for prescription of herbal medicines for a certain disease or its etiology [39–42]. Standardization of diverse pattern identification systems has been inadequate until now, which has made it challenging to apply pattern identification to clinical research on traditional medicine [43–46]. For the findings of clinical studies to be of direct aid in medical decision-making in traditional clinical practice, pattern identification systems used in routine practice need to be accurately reflected in the study design [44, 45]. The present trial was planned in consideration of the target symptoms for herbal formulas, with Western medical methods for participant selection and outcome measurement. This approach is in agreement with a previous proposal for complementation of clinical research methods in traditional medicine [39].

Although the pattern identification and outcome measurement methods of this study are not ideal, they could serve as a reference for planning clinical trials that reflect the characteristics of traditional medicine. The results of this study will provide basic data for the designing of a large-scale trial evaluating the efficacy of GFW for the treatment of climacteric syndrome in women.

### Trial status

Recruitment began in mid-October 2016.

## Additional file

Additional file 1: SPIRIT 2013 checklist. (DOCX 51 kb)

## Abbreviations

AE: Adverse event
ALP: Alkaline phosphatase
ALT: Alanine aminotransferase
ANCOVA: Analysis of covariance
AST: Aspartate aminotransferase
BUN: Blood urea nitrogen
Cr: Creatinine
E2: Estradiol
EDC: Electronic data capture
FAS: Full analysis set
FSH: Follicle-stimulating hormone
GFW: Guizhifulingwan
GMP: Good manufacturing practice
GTP: Glutamyl transpeptidase
Hb: Hemoglobin
Hct: Hematocrit
HRT: Hormone replacement therapy
Hs-CRP: High sensitivity C-reactive protein
ITT: Intention-to-treat
MRS: Menopause rating scale
PPS: Per-protocol set
RBC: Red blood cell
RCT: Randomized controlled trial
SAS: Strategic Applications Software
SPIRIT: Standard Protocol Items: Recommendations for Interventional Trials
TCM: Traditional Chinese medicine
TKM: Traditional Korean medicine
WBC: White blood cell
WHOQOL: World Health Organization quality of life
